# Supplementary figures and images for: Arginase Inhibition Mitigates Bortezomib-Exacerbated Cardiotoxicity in Multiple Myeloma
Source: Cancers (Basel). 2023 Apr 6;15(7):2191. doi: 10.3390/cancers15072191 (PMC10093116; doi:10.3390/cancers15072191)

## Week 2

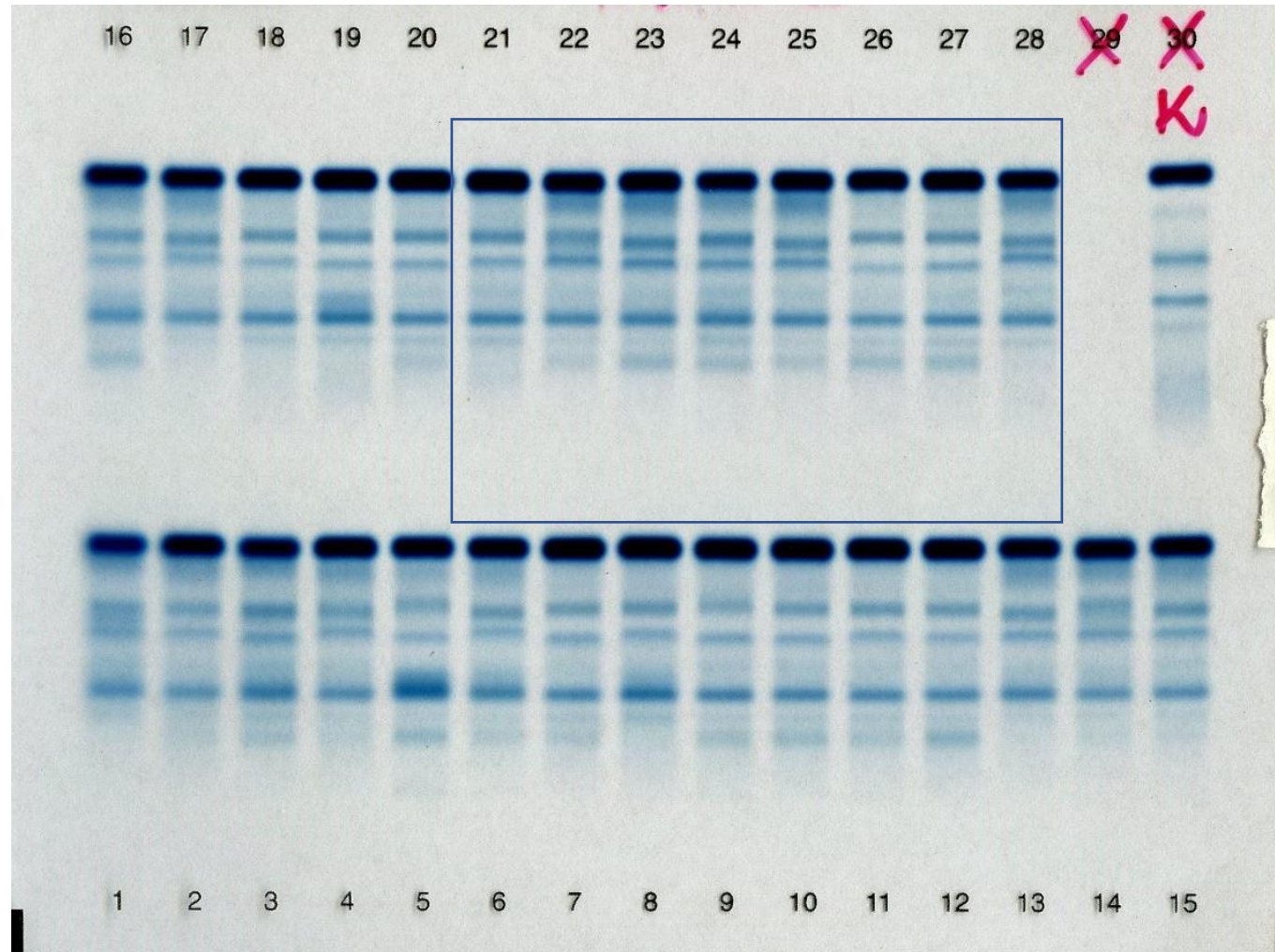

Week 6

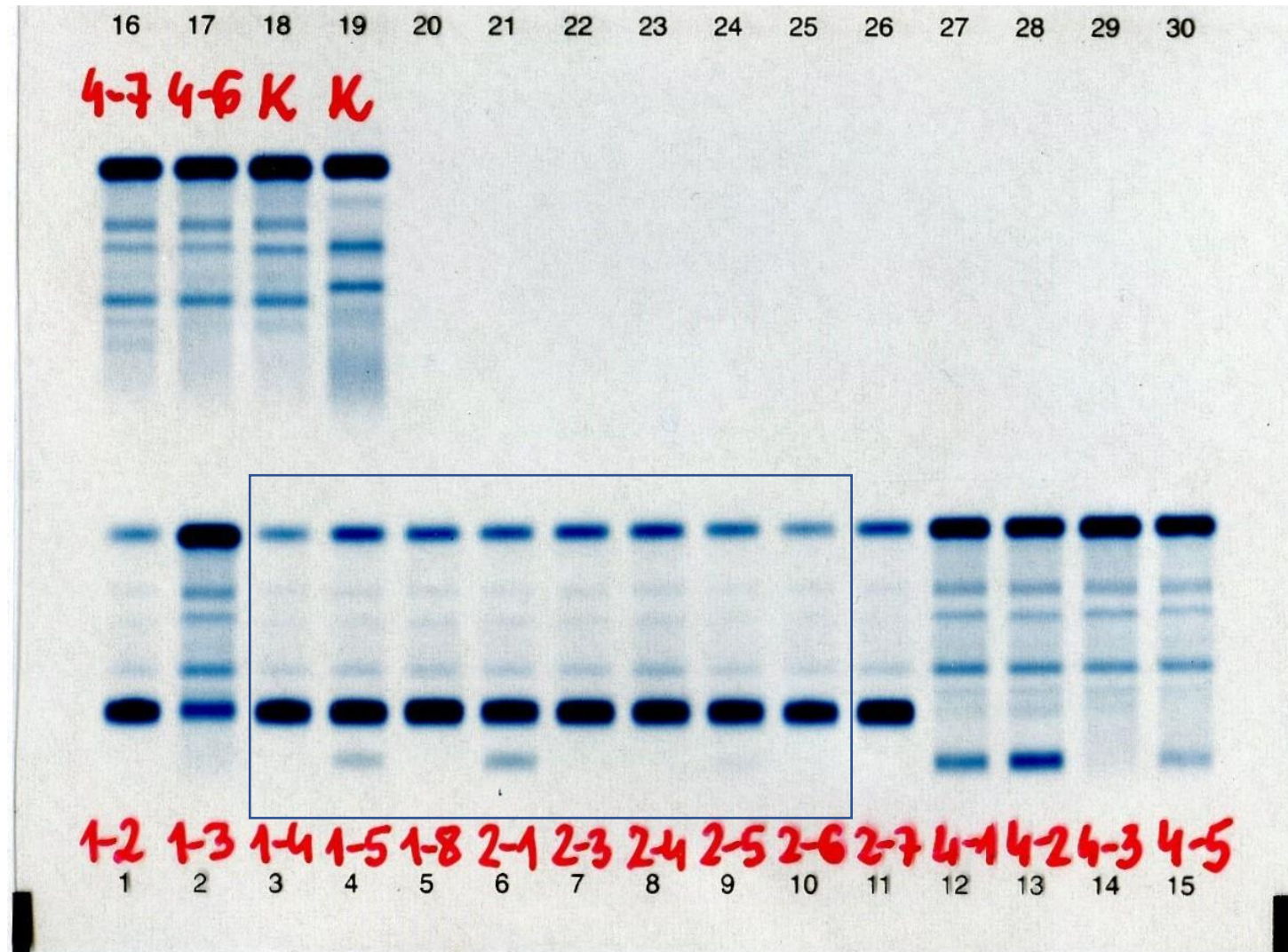

Supplement: Supplementary file 1 [file cancers-15-02191-s001.zip › cancers-2286880-supplementary.pdf]
